# Facilitators and Barriers to Seeking Early Diagnosis and Treatment for Breast Cancer Among Patients Attending a Tertiary Care Hospital

**DOI:** 10.7759/cureus.101401

**Published:** 2026-01-12

**Authors:** Pratibha Singh, Renu Agrawal

**Affiliations:** 1 Department of Community Medicine, Sarojini Naidu Medical College, Agra, IND

**Keywords:** barriers and facilitators, breast cancer care delay, breast self-examination, force field analysis, health‑seeking behavior

## Abstract

Introduction and aim: Breast cancer is the most common cancer among women in India, where a substantial proportion of cases are diagnosed at advanced stages, resulting in preventable morbidity and mortality. This study aimed to identify facilitators and barriers influencing early diagnosis and treatment to inform strategies that promote timely care.

Material and methods: A cross-sectional study was conducted from 2023 to 2025 in the Departments of Community Medicine and Radiotherapy at Sarojini Naidu Medical College, Agra. A total of 95 women aged ≥18 years registered for anti-cancer management were enrolled using consecutive sampling. Data were collected through a pretested semi-structured questionnaire and review of medical records. Factors influencing healthcare-seeking behavior were examined using the social ecological model and force field analysis. Statistical analysis included bivariate comparisons and multivariate logistic regression to identify independent predictors of delay.

Results: Most participants were middle-aged, married, housewives (75.8%), and from low-income households. Significant factors associated with delay included first point of care, primary barrier type, cultural/regional beliefs, and use of indigenous medicine (p<0.05). Major barriers were emotional distress (58.9%), lack of personal transport (66.3%), belief that symptoms would resolve (41.1%), social stigma (40%), and financial or administrative constraints. Facilitators included healthcare provider recommendations (58.9%), government awareness programs (65.3%), family support (62.1%), and preference for female doctors. Awareness of breast cancer screening and breast self-examination practices was significantly associated with reduced delay (p<0.01). Multivariate analysis identified illiteracy (OR: 5.2) and being a housewife (OR: 9.7) as independent predictors of delay.

Conclusion: Delays in breast cancer care are strongly influenced by educational status, gender roles, and socio-cultural beliefs. Enhancing women’s autonomy, expanding awareness initiatives, and strengthening health system support are critical to promoting early diagnosis and improving outcomes.

## Introduction

Breast cancer is the most common cancer among women globally and remains a major contributor to cancer-related mortality, accounting for approximately 11.7% of all new cancer cases worldwide [1]. In India, it has become a significant public health concern, with an estimated 162,468 new cases and 87,090 deaths reported in 2018, making the country the third highest in incidence after the United States and China [2]. The increasing burden underscores the need for early diagnosis and timely treatment, which are essential for improving survival rates and quality of life.

Despite advances in diagnostic technologies and therapeutic options, many breast cancer cases in India continue to be diagnosed at advanced stages. The cancer incidence rate in the country increased by nearly 35% from 1991 to 2021, while the mortality rate rose by 46% during the same period [3]. Late detection significantly compromises treatment success and exacerbates the physical, psychological, and financial strain on patients, families, and the healthcare system [4]. Although early detection through screening can substantially improve outcomes, numerous barriers continue to hinder timely access to care.

India faces unique challenges in cancer prevention and control due to its diverse socio-cultural and economic environment. Low health literacy, cultural stigma, gender norms, and reliance on traditional healing systems contribute to delays in seeking medical help [2-5]. Women in rural areas often lack decision-making autonomy and access to screening facilities. In addition, financial limitations, weak referral pathways, and shortages of trained healthcare providers further impede early diagnosis and treatment [6].

Globally, the World Health Assembly (WHA70.12, 2017) has emphasized the urgency of addressing non-communicable diseases, including cancer, with a target to reduce premature mortality by one-third by 2030 [1]. In alignment with this, the Government of India launched the National Programme for Prevention and Control of Non-Communicable Diseases (NP-NCD) and introduced operational guidelines in 2016 for population-based screening of breast, cervical, and oral cancers [7,8], recommending screening for individuals aged 30-65 years by trained Auxiliary Nurse Midwives at sub-centers [9].

While mammography remains the gold-standard screening tool in high-resource settings, its limited availability and high cost restrict its widespread use in India. Clinical breast examination (CBE), performed by trained health workers, has demonstrated effectiveness as a low-cost early detection strategy in low- and middle-income countries [10,11]. However, implementation challenges persist due to inadequate infrastructure, administrative barriers, insufficient training, and weak follow-up mechanisms, compounded by interstate disparities in resources and political commitment [6]. This study aimed to identify the facilitators and barriers influencing early diagnosis and treatment of breast cancer among patients in India, with the goal of informing targeted interventions to promote early detection, reduce delays in care, and ultimately lower mortality.

## Materials and methods

Study design, setting, and population

This study was conducted as an observational cross-sectional study in the Department of Community Medicine and the Department of Radiotherapy at Sarojini Naidu Medical College, Agra. The study was carried out over a period from 2023 to 2025. The study population included all the females aged 18 years and above attending and registered for anti-cancer management of breast cancer.

Inclusion and Exclusion Criteria

Women aged 18 years and above who were willing to participate and able to respond in person to the study questionnaire were included in the study, while patients who did not provide consent, as well as terminally ill patients and those with co-existing psychiatric illnesses, were excluded.

Sample Size

The sample size was calculated based on the National Family Health Survey (NFHS-5) (2019-21), which reported a 1.2% prevalence of breast cancer screening among urban women [12]. Accordingly, the parameters used for sample size calculation were p = 1.2% (0.012), q = 1-p (0.988), an allowable error (e) of 2.5% (0.025), and a Z value of 1.96 corresponding to a 95% confidence level. The formula used was:

\[ n = \frac{Z^2 \, p \, q}{e^2} \]

Substituting the values:

\[ n = \frac{(1.96)^2 \times 0.012 \times 0.988}{(0.025)^2} \approx 75. \]

After adding a 10\% non-response rate, the adjusted minimum required sample size was:

\[ n_{\text{adj}} = 75 + (0.10 \times 75) = 82.5 \approx 84. \]

Thus, a final sample size of 95 participants was included.

Operational Definitions

Facilitators: Facilitators were defined as factors that enhanced the likelihood of women engaging in breast cancer screening or seeking early diagnosis and treatment. These included enabling influences at the individual, interpersonal, community, or health system level that supported timely healthcare-seeking behavior [13].

Barriers: Barriers were defined as factors that hindered or delayed women from accessing breast cancer screening, diagnosis, or treatment. These included financial constraints, environmental limitations, physical impairments, negative attitudes or behaviors of healthcare providers, lack of knowledge, and psychosocial challenges that adversely affected timely care-seeking [14].

Data Collection

Data were collected using a pretested semi-structured questionnaire administered through face-to-face interviews in the outpatient department while patients waited for their consultations. The questionnaire captured information on socio-demographic characteristics, health history related to breast symptoms, previous healthcare-seeking behavior, and factors that influenced access to timely diagnosis and treatment. It mainly focused on identifying facilitators and barriers influencing early diagnosis and treatment of breast cancer. The questions were developed by the researcher under the guidance of supervisors, while several items were adapted from previously published studies and modified to suit the local context. A pilot study was conducted beforehand to assess clarity, feasibility, and cultural appropriateness, and necessary adjustments were made based on the feedback received.

In addition to interviews, health records were reviewed to obtain clinical details, such as the stage of breast cancer at diagnosis, date and time of first presentation, duration of symptoms prior to seeking care, and tumor size at presentation. The methodology integrated concepts from force field analysis to understand the interplay between enabling factors and obstacles affecting early diagnosis. The conceptual orientation was guided by the social-ecological model-based framework proposed by Saldaña-Téllez et al., which allowed classification of these factors at individual, interpersonal, organizational, socio-cultural, and health policy levels [14]. Participants who met the inclusion criteria were enrolled consecutively until the required sample size was achieved, ensuring systematic and comprehensive data collection throughout the study period.

## Results

The socio-demographic profile of the participants shows that the majority were middle-aged women: 26.3% (n=25) aged 30-40 years and 25.3% (n=24) aged 40-50 years, whereas only 5.3% (n=5) were younger than 20 years. Most participants were housewives, i.e., 75.8% (n=72), reflecting a predominantly non-working female population. Educational status varied, with half of the participants, 50.5% (n=48), educated up to high school, while 26.8% (n=22) had higher education (graduate or postgraduate), and only 3.2% (n=3) were illiterate. A large proportion were married, 70.5% (n=67), with smaller percentages being unmarried, separated, or widowed. Regarding husbands’ occupations, agriculture was the most common at 35.8% (n=34), followed by private employment at 17.9% (n=17). Monthly household income showed that more than one-third, 36.8% (n=35), earned less than ₹10,000, and another 31.6% (n=30) earned below ₹20,000, indicating that the majority belonged to lower-income groups. Overall, the demographic profile reflects a population that is largely middle-aged, married, lower-income, and moderately educated.

**Figure 1:**
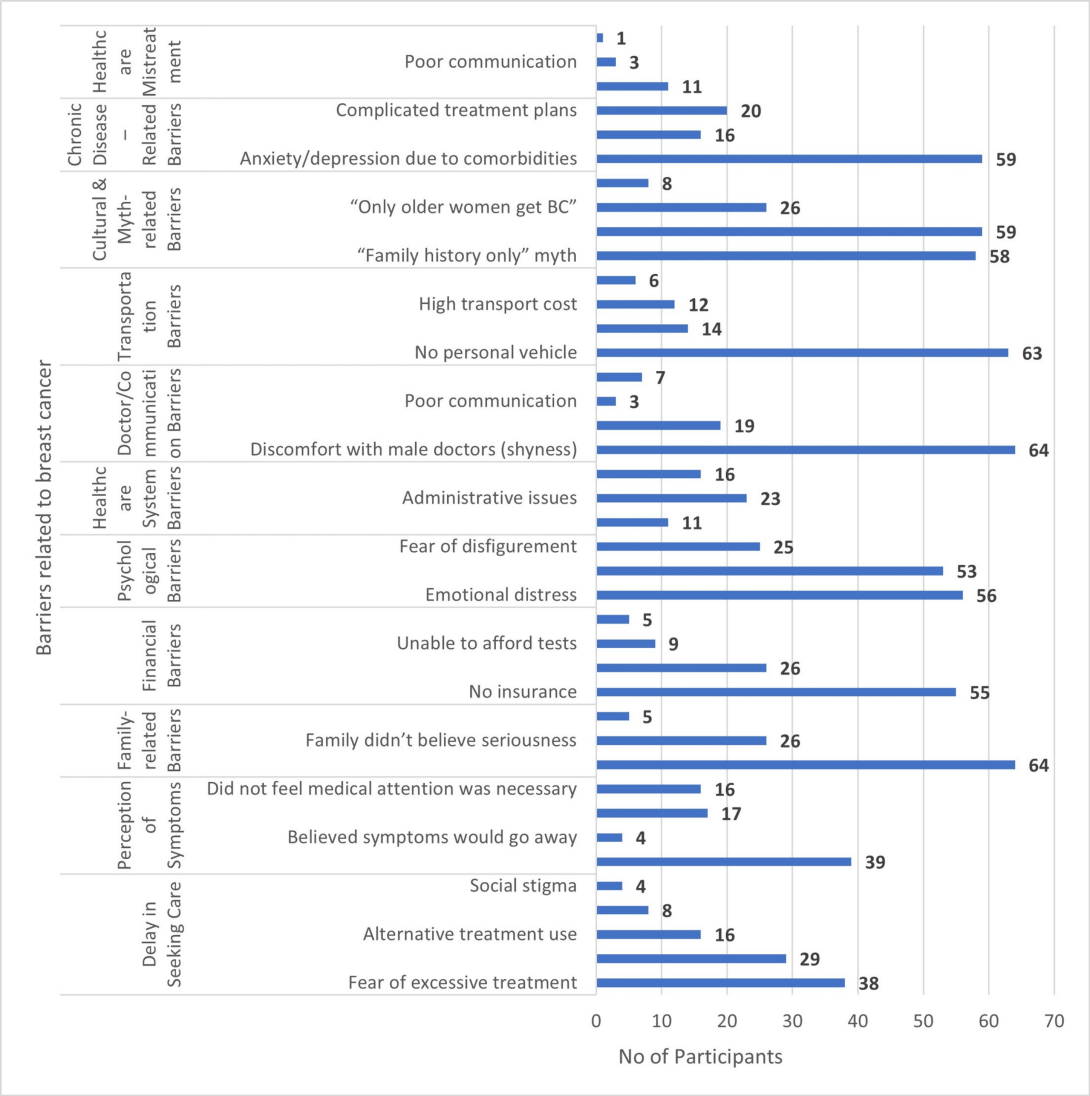
Figure showing barriers to breast cancer care. BC: breast cancer

The most frequently reported barriers were emotional distress (n=56), lack of personal vehicle (n=63), belief that symptoms would resolve on their own (n=39), and social stigma (n=38). Financial constraints, such as inability to afford tests, lack of insurance, and high transport costs, were also common, along with administrative issues and fear of disfigurement (Figure 1).

**Figure 2:**
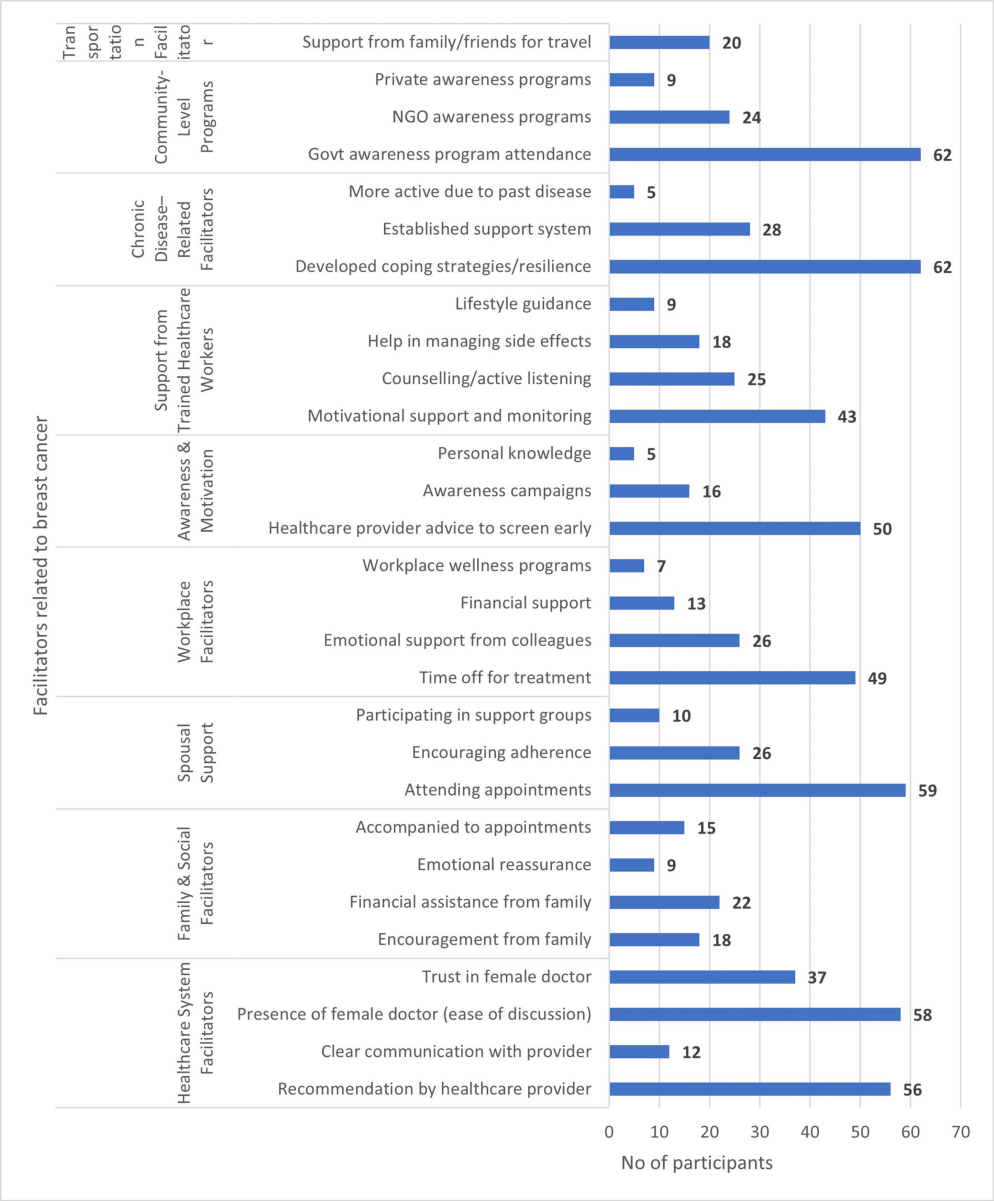
Figure showing facilitators to breast cancer care. NGO: non-governmental organization

Key facilitators included recommendations by a healthcare provider (n=56), government awareness programs (n=62), established support systems and coping strategies (n=62), encouragement and accompaniment from family (n=37 and 59, respectively), and trust/ease of discussion with female doctors (n=58). Workplace flexibility (time off for treatment) and financial support from family or colleagues further enabled ongoing care (Figure 2).

Women with a delay in seeking treatment did not differ significantly from those without a delay in most fears, concerns, family barriers, or financial difficulties. However, delay was significantly associated with first point of care, primary barrier, and reason for discomfort as follows: delayed patients more often first visited primary care/district hospitals, reported non-emotional barriers (cost, waiting time, transport, lack of information), and cited cultural/regional beliefs as reasons for discomfort (all p<0.05) (Table 1).

**Table 1:** Association of psychosocial, family, and healthcare-related barriers with delay in seeking breast cancer treatment. BC: breast cancer

Variables		Delay in seeking treatment		Chi-square	p-Value
		No, n (%)	Yes, n (%)		
Aspect feared	Death	18 (58.1)	35 (54.7)	3.43	0.329
	Disfigurement	10 (32.3)	15 (23.4)		
	Social stigma	1 (3.2)	10 (15.6)		
	Other	2 (6.5)	4 (6.3)		
Concern	Cost	5 (16.1)	18 (28.1)	6.85	0.144
	Side effects	8 (25.8)	5 (7.8)		
	Duration	2 (6.5)	5 (7.8)		
	Effectiveness	13 (41.9)	32 (50.0)		
	Impact on daily life	3 (9.7)	4 (6.3)		
Family barrier	Financial issues	18 (58.1)	46 (71.9)	3.12	0.210
	Did not believe seriousness	12 (38.7)	14 (21.9)		
	Social stigma	1 (3.2)	4 (6.3)		
Fear after diagnosis	Losing loved ones	20 (64.5)	30 (46.9)	3.20	0.202
	Being a burden	7 (22.6)	26 (40.6)		
	Not receiving adequate support	4 (12.9)	8 (12.5)		
Financial difficulty	No health insurance	18 (58.1)	37 (57.8)	5.59	0.133
	Income loss due to BC	11 (35.5)	15 (23.4)		
	Medication cost	2 (6.5)	3 (4.7)		
	Unable to afford tests	0 (0.0)	9 (14.1)		
First point of care	Primary care physician	12 (38.7)	35 (54.7)	11.52	0.009
	Medical college	4 (12.9)	10 (15.6)		
	Specialist	8 (25.8)	2 (3.1)		
	District hospital	7 (22.6)	17 (26.6)		
Primary barrier	Emotional distress	25 (80.6)	31 (48.4)	9.98	0.041
	High treatment cost	2 (6.5)	11 (17.2)		
	Long waiting time	1 (3.2)	10 (15.6)		
	Lack of transportation	3 (9.7)	9 (14.1)		
	Lack of information	0 (0.0)	3 (4.7)		
Reason for discomfort	Personal shyness	23 (74.2)	41 (64.1)	12.25	0.007
	Cultural/regional belief	2 (6.5)	17 (26.6)		
	Lack of rapport/trust	5 (16.1)	1 (1.6)		
	History of trauma/negative experiences	1 (3.2)	5 (7.8)		

Most transport-related factors, breast cancer myths/beliefs, timing of treatment/referral, and administrative or insurance barriers were similar between women with and without delay in seeking treatment (all p>0.05). The only significant difference was in the type of indigenous medicine used (p=0.010) as follows: women with delay more frequently reported using homeopathy (64.1% vs. 32.3%), whereas those without delay more often reported yoga (35.5% vs. 10.9%), indicating a different pattern of alternative therapy use between the groups (Table 2).

**Table 2:** Association of travel, health-system factors, myths, and indigenous medicine use with delay in seeking breast cancer treatment. BC: breast cancer

Variables		Delay in seeking treatment		Chi-square	p-Value
		No, n (%)	Yes, n (%)		
Travel mode	Public transportation	22 (71.0)	43 (67.2)	0.82	0.663
	Personal vehicle	4 (12.9)	6 (9.4)		
	Family/friends	5 (16.1)	15 (23.4)		
Travel distance	<5 miles	5 (16.1)	15 (23.4)	0.67	0.715
	5-10 miles	18 (58.1)	34 (53.1)		
	>20 miles	8 (25.8)	15 (23.4)		
Difficulty	Lack of personal vehicle	19 (61.3)	44 (68.8)	0.85	0.839
	Physical limitations	2 (6.5)	4 (6.3)		
	Limited public transport	6 (19.4)	8 (12.5)		
	High transport cost	4 (12.9)	8 (12.5)		
Myth encountered	Only with family history	18 (58.1)	40 (62.5)	2.86	0.240
	Only older women get BC	7 (22.6)	19 (29.7)		
	Wearing a bra causes BC	6 (19.4)	5 (7.8)		
Belief encountered	BC is a punishment	19 (61.3)	40 (62.5)	5.13	0.077
	You can’t have children	5 (16.1)	19 (29.7)		
	Other belief	7 (22.6)	5 (7.8)		
Time taken to start treatment	1-4 weeks	8 (25.8)	14 (21.9)	0.77	0.858
	Within 1 week	17 (54.8)	35 (54.7)		
	>1 month	3 (9.7)	5 (7.8)		
	Immediately	3 (9.7)	10 (15.6)		
Time to referral to a specialist	Same day	7 (22.6)	17 (26.6)	1.00	0.801
	1 week	21 (67.7)	39 (60.9)		
	2-4 weeks	2 (6.5)	7 (10.9)		
	>1 month	1 (3.2)	1 (1.6)		
Reasons for delay	Personal circumstances	17 (54.8)	39 (60.9)	1.79	0.409
	Insurance related	4 (12.9)	12 (18.8)		
	Administrative issues	10 (32.3)	13 (20.3)		
Indigenous medicine consulted	Yoga	11 (35.5)	7 (10.9)	11.37	0.010
	Ayurveda	9 (29.0)	13 (20.3)		
	Homeopathy	10 (32.3)	41 (64.1)		
	Unani	1 (3.2)	3 (4.7)		
Factors influencing use of indigenous medicine	Cultural beliefs	25 (80.6)	46 (71.9)	4.26	0.119
	Previous positive experiences	6 (19.4)	10 (15.6)		
	Distrust of conventional medicine	0 (0.0)	8 (12.5)		

Most relational and support-related factors - past mistreatment by healthcare professionals, type of mistreatment, family and spousal support, workplace support, preference for female doctors, enabling factors, and type of first consulted provider - did not differ significantly between women with and without delay in seeking treatment (all p>0.05). Although not statistically significant, women with delay tended to report family support (as an enabling factor) more often and were slightly more likely to first consult a surgeon, while those without delay more often consulted a primary care physician or radiologist (Table 3).

**Table 3:** Association of interpersonal support, healthcare experiences, and care-seeking pathways with delay in seeking breast cancer treatment.

Response		Delay in seeking treatment		Chi-square	p-Value
		No, n (%)	Yes, n (%)		
Ever mistreated/disrespected by a healthcare professional	Yes	4 (12.9)	7 (10.9)	0.079	0.779
	No	27 (87.1)	57 (89.1)		
Types of mistreatments experienced	Dismissive attitudes	1 (25.0)	0 (0.0)	3.592	0.166
	Inadequate explanation of options	3 (75.0)	4 (57.1)		
	Other dismissive/negative behavior	0 (0.0)	3 (42.9)		
Family support received adequately	Encouragement to seek treatment	20 (64.5)	29 (45.3)	3.566	0.312
	Accompanied to appointments	3 (9.7)	12 (18.8)		
	Emotional reassurance	3 (9.7)	6 (9.4)		
	Financial assistance	5 (16.1)	17 (26.6)		
Ways spouse provides emotional support	Encouraging adherence	9 (29.0)	17 (26.6)	1.810	0.405
	Attending appointments	17 (54.8)	42 (65.6)		
	Participating in support groups	5 (16.1)	5 (7.8)		
Workplace support	Emotional support from colleagues	9 (29.0)	17 (26.6)	0.120	0.989
	Time off for treatment	16 (51.6)	33 (51.6)		
	Wellness programs	2 (6.5)	5 (7.8)		
	Financial assistance	4 (12.9)	9 (14.1)		
Availability of female doctors as a factor	Ease of discussing breast issues	18 (58.1)	40 (62.5)	0.173	0.678
	Trust in female doctors	13 (41.9)	24 (37.5)		
Factors enabling you to seek healthcare	Healthcare recommendation	19 (61.3)	37 (57.8)	6.053	0.109
	Clear communication	6 (19.4)	6 (9.4)		
	Family support	2 (6.5)	16 (25.0)		
	Insurance coverage	4 (12.9)	5 (7.8)		
First consulted for specific healthcare	Primary care physician	21 (67.7)	36 (56.3)	4.964	0.174
	Radiologist	4 (12.9)	3 (4.7)		
	Surgeon	5 (16.1)	19 (29.7)		
	Other	1 (3.2)	6 (9.4)		

Attendance at government or NGO awareness programs, motivation to seek early diagnosis (from family, providers, or self-knowledge), and support from trained healthcare workers were all common in both the <6-month and ≥6-month delay groups. Although numerically more women with longer delays had attended government programs or received motivational support, the differences were not statistically significant (all p>0.05), suggesting that awareness and facilitation alone did not reliably prevent delay.

Women who had ever performed breast self-examination (BSE) and those who had heard about breast cancer screening were more often in the "no delay" group, and these associations were statistically significant (p<0.01). Frequency of BSE (weekly, monthly, occasional) also differed significantly between delayed and non-delayed groups (p=0.009), while source of information (doctor, media, family/friends) showed no significant association (p=0.165).

Although not statistically significant (p=0.087), participation in breast cancer awareness programs showed a trend toward earlier healthcare seeking. Women attending government-led programs (80.6%; n=25) were more likely to seek treatment within six months, while those exposed to NGO initiatives (31.3%; n=20) were more common among delayed cases. The main motivators for seeking early diagnosis were healthcare provider advice (58.1%; n=18) and family encouragement (25%; n=16). Among healthcare worker roles, motivational support (43.8%; n=28) and guidance in managing side effects (23.4%; n=15) were more frequent among delayed participants. These findings suggest that structured, government-supported awareness programs and active healthcare facilitation can positively influence early diagnosis and timely treatment in breast cancer patients (Table 4).

**Table 4:** Association between socio-demographic variables and delay (system delay vs. patient delay).

Variables		Delay		Chi-square	p-Value
		System delay, n (%)	Patient delay, n (%)		
Age group (years)	1-20	3 (3.6)	2 (16.7)	5.188	0.269
	20-30	19 (22.9)	3 (25.0)		
	30-40	23 (27.7)	2 (16.7)		
	40-50	20 (24.1)	4 (33.3)		
	>50	18 (21.7)	1 (8.3)		
Occupation	Housewife	60 (72.3)	12 (100.0)	4.388	0.036
	Working	23 (27.7)	0 (0.0)		
Education level	Illiterate	1 (1.2)	2 (16.7)	15.811	0.007
	Primary/high school	5 (6.0)	3 (25.0)		
	High school	46 (55.4)	2 (16.7)		
	Interschool	12 (14.5)	2 (16.7)		
	Graduate	10 (12.0)	2 (16.7)		
	Postgraduate	9 (10.8)	1 (8.3)		
Marital status	Married	59 (71.1)	8 (66.7)	1.830	0.608
	Unmarried	13 (15.7)	3 (25.0)		
	Separated	7 (8.4)	0 (0.0)		
	Widow	4 (4.8)	1 (8.3)		
Monthly income (INR)	<10,000	32 (38.6)	3 (25.0)	1.150	0.765
	10,000-20,000	26 (31.3)	4 (33.3)		
	20,000-30,000	18 (21.7)	4 (33.3)		
	30,000-40,000	7 (8.4)	1 (8.3)		

Multivariate logistic regression showed that low education and being a housewife were the only significant independent predictors of delay in seeking treatment. Illiterate women had approximately five-fold higher odds of diagnostic delay compared to literate women (adjusted OR: 5.2; 95% CI: 1.54-17.4; p=0.007). Housewives had markedly elevated odds of delay compared to working women (adjusted OR: 9.71; 95% CI: 0.55-17.69; p=0.036).

In contrast, age, marital status, income, prior breast disease, family history, awareness of screening, participation in awareness programs, healthcare worker involvement, transport difficulties, travel distance, and mode of travel were not independently associated with delay (all p>0.05), suggesting that structural and informational factors alone are insufficient without genuine empowerment and decision-making capacity (Table 5).

**Table 5:** Multivariate logistic regression analysis of socio-demographic and clinical factors associated with type of delay. *P<0.05 was statistically significant. F/H/O: family history of; H/O: history of

Variables	Category (reference)	OR	95% CI	p-Value
Age (years)	<40 vs. ≥40	0.62	0.18-2.14	0.269
Occupation	Housewife vs. working	9.71	0.55-17.69	0.036*
Education level	Illiterate vs. literate	5.20	1.54-17.4	0.007*
Marital status	Married vs. others	0.82	0.33-2.05	0.608
Monthly income (INR)	<10,000 vs. ≥10,000	0.88	0.27-2.86	0.765
H/O breast disease	Yes vs. no	1.02	0.131-5.71	0.336
F/H/O breast cancer	Yes vs. no	0.58	0.06-5.42	0.632
Time to see a doctor	>6 months vs. <6 months	0.64	0.18-2.32	0.475
Awareness of breast cancer screening	Yes vs. no	0.92	0.25-3.35	0.900
Role of trained healthcare worker	Active vs. none	0.86	0.25-2.90	0.950
Attendance in awareness program	Government vs. others	1.75	0.36-8.40	0.472
Spousal emotional support	Present vs. absent	1.15	0.27-4.84	0.398
Family support	Encouragement vs. none	1.92	0.42-8.79	0.715
Cannot have children after cancer	Yes vs. no	1.56	0.39-6.19	0.333
Other/unspecified myths	Yes vs. no	2.52	0.58-10.9	0.230
Transportation difficulty	Present vs. none	1.89	0.63-5.68	0.262
Travel distance (>10 miles)	Yes vs. ≤10 miles	1.37	0.30-6.22	0.721
Mode of travel (public)	Public vs. others	0.71	0.18-2.75	0.671

## Discussion

In this study of 95 breast cancer patients, the socio-demographic profile of predominantly middle-aged, married, lower-income housewives (26.3% {n=25} aged 30-40 years, 25.3% {n=24} aged 40-50 years, 75.8% {n=72} housewives, 36.8% {n=35} earning less than ₹10,000, and 31.6% {n=30} less than ₹20,000) broadly aligns with other Indian data on vulnerable groups. Kaur et al. reported that most women were more than 50 years old, and nearly half (47.4%) were illiterate, with low education and low socio-economic status significantly increasing perceived barriers [15]. Although only 3.2% (n=3) of our cohort were illiterate, low education level still independently predicted delay (OR: 5.2; 95% CI: 1.54-17.4; p=0.007), indicating that educational disadvantage strongly impedes timely care even in relatively well-educated samples.

Being a housewife also significantly predicted delay (p=0.036), consistent with Kaur et al., who found that prioritizing family duties over personal health (70.2%) and lower socio-economic class (OR: 1.43; p=0.001) heightened barriers [15]. Qualitative findings by Nandini et al. similarly highlighted financial constraints, negligence, and weak family support as key reasons for late presentation [16]. Together with our results, these studies underscore gendered roles, economic dependence, and limited autonomy as structural drivers of delay.

Care pathways in our study showed that delayed patients more often first consulted primary care/district hospitals and faced non-emotional barriers such as cost, waiting time, transport, and poor information. In contrast, Kumar et al. observed that 70.6% first visited private providers, with a median overall delay of 203 days, largely due to prolonged treatment delay (median 130 days), multiple non-specialist consultations, and misclassification of severity [17]. Despite these contextual differences, both studies highlight the importance of provider preparedness and efficient referral in reducing delays.

Barriers in our cohort, such as emotional distress (56 women), belief that symptoms would resolve (39), social stigma (38), and financial problems, mirror the fear, misconceptions, stigma, and financial hardship described by Kaur et al., Nandini et al., and Kumar et al. additionally reported high fatalistic beliefs and poor knowledge of screening and early diagnosis [15-17]. In our study, cultural/regional beliefs were significantly linked to delay, echoing the "rigid social customs and beliefs" identified by Palaniraja et al. among community health workers (CHWs) [18].

Awareness programs and CHW support, while common in both delay and no-delay groups, did not significantly reduce delay, similar to Palaniraja et al.’s observation that CHWs face programmatic and infrastructural constraints [18]. By contrast, individual preventive practices were protective - ever-performing breast self-examination (BSE), awareness of screening, and higher BSE frequency were significantly associated with no delay (p<0.01 and p=0.009), consistent with Kathrikolly et al.'s emphasis on translating knowledge into action through culturally sensitive, supportive strategies [19,20].

Finally, the greater use of homeopathy among delayed women (64.1% vs. 32.3%) and yoga among non-delayed women (35.5% vs. 10.9%) provides novel quantitative insight into alternative therapy patterns, complementing earlier qualitative descriptions [17-19]. Overall, our findings reinforce that education, economic autonomy, and culturally attuned system-level facilitation are more critical to preventing delay than awareness activities alone.

A key strength of this study is its comprehensive assessment of multi-level barriers and facilitators, including socio-demographic factors, health-system issues, emotional and cultural barriers, indigenous therapy use, awareness activities, and preventive practices such as BSE. The use of multivariate logistic regression to identify independent predictors of delay and the generation of context-specific quantitative data from a real-world tertiary care setting add meaningful value to the predominantly qualitative Indian literature on breast cancer delay.

However, the study has some limitations. Its single-center design and relatively small sample size (n=95) may limit generalizability to other regions and healthcare settings. The cross-sectional design precludes establishing causality between identified determinants and delay, and reliance on self-reported data introduces the risk of recall and social desirability bias, especially regarding symptom onset, time intervals, and participation in awareness programs.

## Conclusions

Our study shows that delays in breast cancer treatment are influenced more by socio-demographic and structural factors, such as low education, being a housewife, financial dependence, cultural beliefs, and use of indigenous therapies, than by lack of awareness alone. Although many women attended awareness programs and interacted with health workers, these factors did not independently reduce delay. In contrast, preventive behaviors like breast self-examination and prior knowledge of screening were strongly associated with timely care.

These findings highlight the need for interventions that enhance women’s autonomy, financial stability, and shared decision-making, while ensuring culturally sensitive counseling and stronger primary-level referral pathways. Programs combining government-led awareness activities with trained community health workers and survivor-led navigation may improve early care-seeking. Future research should use larger multi-centric designs to confirm these determinants and assess targeted empowerment approaches. Exploring digital tools, community navigators, and social protection mechanisms may further help reduce delays in diagnosis and treatment.
